# Dynamic *in vitro* culture of cryopreserved-thawed human ovarian cortical tissue using a microfluidics platform does not improve early folliculogenesis

**DOI:** 10.3389/fendo.2022.936765

**Published:** 2022-07-29

**Authors:** Julieta S. Del Valle, Vanessa Mancini, Maitane Laverde Garay, Joyce D. Asseler, Xueying Fan, Jeroen Metzemaekers, Leoni A. Louwe, Gonneke S. K. Pilgram, Lucette A. J. van der Westerlaken, Norah M. van Mello, Susana M. Chuva de Sousa Lopes

**Affiliations:** ^1^ Department of Anatomy and Embryology, Leiden University Medical Center, Leiden, Netherlands; ^2^ Department of Obstetrics and Gynaecology, Amsterdam University Medical Center (UMC), Amsterdam, Netherlands; ^3^ Department of Gynaecology, Leiden University Medical Center, Leiden, Netherlands; ^4^ Ghent-Fertility and Stem Cell Team (G-FAST), Department of Reproductive Medicine, Ghent University Hospital, Ghent, Belgium

**Keywords:** folliculogenesis, human, follicular growth, microfluidics, ovarian cortical tissue, cryopreservation, secondary follicle, fertility preservation

## Abstract

Current strategies for fertility preservation include the cryopreservation of embryos, mature oocytes or ovarian cortical tissue for autologous transplantation. However, not all patients that could benefit from fertility preservation can use the currently available technology. In this regard, obtaining functional mature oocytes from ovarian cortical tissue *in vitro* would represent a major breakthrough in fertility preservation as well as in human medically assisted reproduction. In this study, we have used a microfluidics platform to culture cryopreserved-thawed human cortical tissue for a period of 8 days and evaluated the effect of two different flow rates in follicular activation and growth. The results showed that this dynamic system supported follicular development up to the secondary stage within 8 days, albeit with low efficiency. Surprisingly, the stromal cells in the ovarian cortical tissue were highly sensitive to flow and showed high levels of apoptosis when cultured under high flow rate. Moreover, after 8 days in culture, the stromal compartment showed increase levels of collagen deposition, in particular in static culture. Although microfluidics dynamic platforms have great potential to simulate tissue-level physiology, this system still needs optimization to meet the requirements for an efficient *in vitro* early follicular growth.

## Introduction

According to the World Health Organization (WHO), infertility is a global disease affecting more than 186 million people mainly from developed countries (https://www.who.int/news-room/fact-sheets/detail/infertility). While infertility is often associated with defects in the male or female reproductive systems, it may also result from other factors such as lifestyle, stress or the progressively older age of the female partner at conception (1). In the last decades, innovative techniques in medically assisted reproduction, such as *in vitro* fertilization, have helped an increasingly large group of patients to overcome infertility (2). For patients exposed to either gonadotoxic treatment, predisposed to premature ovarian insufficiency or undergoing surgical removal of both ovaries, there are several options for fertility preservation, that include the cryopreservation of mature oocytes to be used during medically assisted reproduction, or ovarian tissue cryopreservation for autografting (3, 4). However, for patients such as pre-pubertal oncological patients with hematological malignancies, none of the currently available strategies are adequate due to the absence of mature oocytes and the high risk of reintroducing cancer cells after autografting (5–7). For this growing group of patients, that undergoes ovarian tissue cryopreservation to preserve the ovarian follicular reserve and survives to adulthood, obtaining mature oocytes from cortical ovarian tissue cultured *in vitro*, could be considered a safe alternative method in the context of fertility preservation (8).

In humans, folliculogenesis is a complex process that starts with the formation of primordial follicles before birth (9). After birth, small groups of primordial follicles, formed by one primary oocyte arrested in dictyate (diplotene stage from meiotic prophase I) surrounded by a single layer of flat granulosa cells and a layer of basement membrane, undergo follicular activation, as the granulosa cell layer adopts a cuboidal morphology and the primordial follicle transits to primary follicle (10, 11). During follicular growth, the cuboidal granulosa cells undergo proliferation, forming several cell layers around the oocyte (secondary follicle) and start accumulating follicular fluid in a growing central cavity or antrum (antral follicle). In mono-ovulatory species such as humans, typically one antral follicle becomes dominant, growing from 5 up to 20 millimeters in diameter and undergoing ovulation (12–14), whereas the other antral follicles regress through atresia (15). In the ovulatory follicle, the oocyte resumes meiosis and arrests in metaphase II, the competent stage for fertilization. It is only after fertilization that the oocyte completes meiosis with the extrusion of the second polar body. During adulthood, the process from follicular activation until ovulation requires several months (16, 17).

The development of robust clinically-applicable protocols remains challenging despite ongoing efforts to culture cryopreserved (or fresh) ovarian cortical tissue *in vitro* through *in vitro* follicular activation, *in vitro* follicular growth and oocyte *in vitro* maturation to ultimately obtain mature oocytes (metaphase II) that could be fertilized (18). One of the main reasons for this is the lack of knowledge regarding the control mechanisms that regulate follicular activation and growth (19) due to the relative rarity of healthy human ovarian tissue available for scientific research. Several studies have reported the formation of antral follicles *in vitro* from cultured pre-antral/secondary follicles present in human ovarian cortical tissue (20–24). However, the follicular population that shows the highest survival rate after cryopreservation and thawing procedures are the primordial follicles (25). To date, only two groups have reported the growth and maturation to metaphase II oocytes starting from unilaminar (primordial/primary) follicles present in fresh human ovarian cortical tissue, by using a multistep static culture system (26, 27), but attempts to fertilize such obtained metaphase II oocytes have not been reported.

The traditional static ovarian culture system is a robust and simple system that mimics the complexity of physiological conditions only to a limited extend. This system allows the accumulation in the culture media of compounds released by the tissue that may act as paracrine signals for cell growth and survival. However, due to follicular growth, the culture media needs to be replaced with some frequency to provide the cells with fresh nutrients and remove waste products. This manipulation disturbs the medium composition and consequently the cellular state. Dynamic culture systems using microwells have the possibility of culturing tissue in a small volume, where the concentration of paracrine factors can reach high values, while making use of a large reservoir of circulating medium and adjustable flow rates that allow a continuous supply of nutrients.

Innovative organ-on-chip models, microfluidic platforms and engineered culture systems are increasing in complexity to recreate the physiological conditions of the reproductive system and stimulate follicular growth and maturation (28, 29). One example of these advanced culture systems is the microfluidics platform EVATAR, whereby several organ modules (ovary, fallopian tube, uterus, cervix and liver) were connected by circulating flow between the organ modules (30). Another example is the transwell-based system that was used to co-culture human fallopian tube epithelium and murine ovarian follicles in two different compartments (31). These innovative tissue culture systems to improve folliculogenesis *in vitro* have shown promising results, but have so far only been used with mouse follicles. Hence, the application of engineered systems to follicles present in cryopreserved-thawed human cortical tissue is urgently needed.

Using a microfluidics platform, our study aims to investigate the effects of dynamic culture conditions on human ovarian cortex to stimulate follicular activation and growth *in vitro*. Cryopreserved-thawed ovarian cortical tissue was cultured in a microfluidic chip in static and dynamic conditions using two different flow rates and the quantity and quality of secondary follicles was investigated after four and eight days in culture. The culture period used was based on the multistep static culture system developed by McLaughlin and colleagues (26), where secondary follicles could be obtained from unilaminar follicles present in human ovarian cortex *in vitro* within 8 days (Step I).

## Materials and methods

### Ethics and patient characteristics

The study was conducted according to the guidelines of the Declaration of Helsinki. The study design was submitted to the Medical Ethical Committee of the LUMC and a letter of no objection was obtained (B18.029) prior to the study. Signed informed consent was obtained from the tissue donors (N=13) undergoing gender-affirming surgery at the VUmc hospital (Amsterdam, Netherlands) and at the Amstelland hospital (Amsterdam, Netherlands). Patient characteristics, such as age, gender-affirming hormone treatment (testosterone-based) and treatment duration prior to gender-affirming surgery are provided in **Table S1**.

### Ovarian cortex isolation, cryopreservation and thawing

Ovaries were processed and cryopreserved using a slow freezing method as described (32). Briefly, the ovaries were bisected and placed 0.9% NaCl solution (B230551, Fresenius Kabi, France). The outer cortex, with a thickness of about 1mm, was separated from the inner cortex and medulla using scalpels (0508, Swann-Morton, UK) and was cut into smaller pieces of 10mm x 5mm. Thereafter, individual ovarian cortex pieces were put into cryovials (126263, Greiner, Netherlands) containing 1ml of cryoprotectant solution [0.1M sucrose (S9378-1KG, Sigma Chemicals, Netherlands) and 1.5M ethylene glycol (102466, Sigma Chemicals, Netherlands) in phosphate-buffered saline (PBS) (14190094, Merck, Germany)]. The tissue was left in the cryoprotectant solution for 30 minutes (min) before starting the slow freezing program performed by a programmable Planer freezer (GDMRV, PLANNER, UK). The freezing protocol applied was the following: 2°C/min to −9°C, 5min of soaking, manual seeding for ice crystal nucleation induction using a cotton swab dipped into liquid nitrogen, 0.3°C/min to −40°C, 10°C/min to −140°C and the cryovials were placed into liquid nitrogen (−196°C) containers for storage.

For thawing, the cryovials were kept in a water bath at 37°C until the medium around the frozen tissue had thawed (3-5min). To remove the cryoprotectant solution, the tissue was washed for 10min at room temperature (RT) with occasional shaking in a solution of 0.75M ethylene glycol and 0.25M sucrose in PBS, followed by a 10min wash in 0.35M sucrose in PBS, and finally a 10min wash in PBS.

### Ovarian cortex culture

Thawed cortical ovarian tissue pieces (N=6 donors) were cut into 1mm × 1mm × 1mm cubes using scalpels in PBS. After thawing (day 0), the cortical cubes were either fixed (n=8-15) or placed in microwells (n=12, 2 cubes per well) from a μ-slide III 3D perfusion plates (80376, Ibidi GmbH, Gräfelfing, Germany) for *in vitro* culture. After closing the open microwells using an adhesive coverslip, the microchannels were filled with culture medium using a 1ml syringe (303172, Dalsup BD, Netherlands) through the inlet ports of the slides (each inlet port is connected to 2 microwells of 30μl volume). The culture medium used was McCoy’s 5a with bicarbonate and 20mM HEPES (22330021, Invitrogen, Paisley, UK) supplemented with 3mM glutamine (25030-024, Invitrogen, Paisley, UK), 0.5% human serum albumin (C1309/490, Alburex20, CSL Behring, UK), 1x penicillin and streptomycin (P4458-100ML, Sigma Chemicals, Netherlands), 0.1% amphotericin B (A2942-20ML, Sigma Chemicals, Netherlands), 1x Insulin-Transferrin-Selenium-Ethanolamine (ITS -X) (51500-056, Invitrogen, Paisley, UK) and 50μg/ml ascorbic acid (A8960, Sigma Chemicals, Netherlands) adapted from McLaughlin and colleagues (McLaughlin et al., 2018). For the first 24 hours, 12μM sphingosine-1-phosphate (S1P) (860492P-1MG, Merk, Netherlands) was added to the media to promote follicular growth (33). Fragments were cultured for four or eight days at 37°C in humidified air and 5% CO_2_.

During static culture, the media was replaced every 2 days by adding 70μl of fresh media through each inlet (connected to 2 microwells in series). In the dynamic culture, each plate was connected to a Ibidi pump system (Ibidi, GmbH, Gräfelfing, Germany). The characteristics of the perfusion set were: tubing with 15cm length and 0.8mm internal diameter (ID) to provide a high flow rate (0.5ml/min) and tubing with 50cm length and 0.5mm ID to provide a low flow rate (0.1ml/min). The fluidic unit was assembled in the hood, transferred to an incubator and connected to the pump system. Up to four fluidic units were connected to the pump in the same experiment. Each unit had two 10ml reservoirs and, for each experiment, 5ml of media was added to each reservoir before starting the experiment.

Fresh ovarian cortex pieces (N=3 donors) were cut into 1mm × 1mm × 1mm cubes using scalpels in PBS and cultured in static condition in 12-well plates (Thermo Fisher Scientific) (n=12 cubes per well) in 500μl culture medium (as above) with or without 1ng/ml recombinant follicle-stimulating hormone (FSH) (F4021-2μg, Sigma Chemicals, Netherlands). Half of the medium was replaced every 2 days. Fragments were cultured for 8 days at 37°C in humidified air and 5% CO_2_.

### Histochemistry, immunofluorescence and TUNEL assay

Freshly collected ovaries as well as fresh or cryopreserved-thawed ovarian cortex tissue samples collected on day 0, day 4, or day 8 after culture were fixed in 4% paraformaldehyde (Merck, Germany) in PBS overnight at 4°C. Thereafter, the tissue was washed overnight in PBS, transferred to 70% ethanol and embedded in paraffin using a Shandon Excelsior tissue processor (Thermo Scientific, Altrincham, UK). After embedding, the tissue was sectioned (5μm) using an RM2065 microtome (Leica Instruments GmbH, Wetzlar, Germany) and the sections stretched onto StarFrost microscope slides (3057-1, Waldemar Knittel, Germany). Hematoxylin and eosin (HE) staining on paraffin sections was performed as previously described (Heeren et al., 2015).

For immunofluorescence, the sections were deparaffinized and treated with Tris–EDTA buffer (10mM Tris, 1 mM EDTA solution, pH 9.0) for 12min at 98°C in a microwave (TissueWave 2, Thermo Scientific). The sections were allowed to cool down, rinsed with PBS (2 × 5min) and 0.05% Tween-20 (822184, Merck, Germany) in PBS (PBST) (5min) and blocked 1 hour with 1% bovine serum albumin (BSA) (A8022-100G, Life Technologies, USA) in PBST at RT in a humidified chamber. After blocking, the primary antibodies diluted in blocking buffer were added and the slides were incubated overnight at 4°C. Subsequently, the slides were rinsed with PBS (2 × 5min) and PBST (5min) and incubated 1 hour with the secondary antibodies diluted in blocking buffer at RT. Next, the slides were rinsed with PBS (2 × 2min), PBST (2min), distilled water (2min) and mounted with Pro-Long Gold (P36930, Life Technologies, USA). Negative controls were obtained by omitting the primary antibodies.

The primary antibodies used were goat anti-FOXL2 (1:200, NB100-1277, Bio-Techne), mouse anti-AMH (1:30, MCA2246, R&D system), rabbit anti-KRT19 (1:100, ab76539, Abcam), rabbit anti-COLIV (1:50, AB748, Abcam) and mouse anti-PCNA (1:100, sc-56, Bio-connect). The secondary antibodies used were Alexa Fluor 488 donkey anti-rabbit IgG (1:500, A21206, Life Technologies), Alexa Fluor 647 donkey anti-goat IgG (1:500, A-21447, Life Technologies) and Alexa Fluor 594 donkey anti-mouse IgG (1:500, A21203, Life Technologies). TUNEL-assay was performed using the *In Situ* Cell Detection Kit FITC (11684817910, Roche, Germany) according to manufacturer’s instructions. The nuclei were stained with 4′,6-diamidino-2-phenyl-indole (DAPI) (1:1000, D1306, Life Technologies, USA) and sections were mounted using Pro-Long Gold.

### Imaging and quantification

Confocal fluorescence images were obtained on a TC SP8 inverted confocal microscope (Leica, Wetzlar, Germany), using a ×40 oil immersion objective and LAS X software (Leica, Wetzlar, Germany), and color adjustments were performed using Fiji (34).

The HE-stained slides were scanned using a Panoramic 250 digital scanner (3DHISTECH Ltd., Budapest, Hungary) and viewed using CaseViewer software (3DHISTECH Ltd., Budapest, Hungary). For the quantification of the follicles, the total number of follicles present in 8 different HE-stained sections, apart 40μm to prevent double-counting, per cortical ovarian cube were counted and pooled per culture condition. To overcome intrinsic differences in follicular number between ovarian cubes, we have summed the number of follicles per cube per donor. In addition, to reduce the variation between donors, instead of comparing the total number of follicles per donor, we have compared the percentage of follicular types present in all cubes per donor and provided the mean percentage between the donors per condition.

The criteria to classify the different follicular stages was: primordial follicles had a single layer of flat granulosa cells; transitional/primary follicles were unilaminar, but contained at least some cuboidal granulosa cells; secondary follicles had at least two layers of granulosa cells and no antrum; antral follicles had five or more layers of surrounding granulosa cells and an antral cavity. Atretic follicles were follicles that contained pyknotic nuclei in the oocyte and granulosa cells, and presented oocyte shrinkage with red-coloration in HE and/or cell detachment from the basement membrane. Early atretic follicles were unilaminar and secondary atretic follicles presented multiple layers of granulosa cells.

To measure the relative fluorescence intensity of TUNEL and COLIV, arbitrary images essentially containing stromal compartment and no secondary follicles per condition (N=6 images from three different donors) were taken using a TC SP8 inverted confocal microscope (Leica) with ×40 oil immersion objective. The fluorescence intensity signal from TUNEL, COLIV and DAPI were measured using Fiji software. The corrected total cell fluorescence (CTCF) was calculated by measuring TUNEL or COLIV fluorescence signal on the whole image area and normalized against the fluorescence intensity signal from DAPI present in the same image.

### Statistical analysis

The results, presented as mean ± standard deviation or mean ± standard error of the mean (SEM), were analyzed with GraphPad Prism v9.0.1 software (Graph Pad Software Inc., California, USA). Statistical significance was determined by using Shapiro-Wilk test for normal distribution followed by multiple t-test (Figure 3A), one-way ANOVA followed by Fisher test( Figure 3D and Figure 6B and 6D) and two-way ANOVA followed by Fisher test (Figure S2D). P_value < 0.05 (*), < 0.01 (**) and < 0.001 (***) were considered statistically significant.

## Results

### Folliculogenesis in transmasculine ovaries

**Figure 1:**
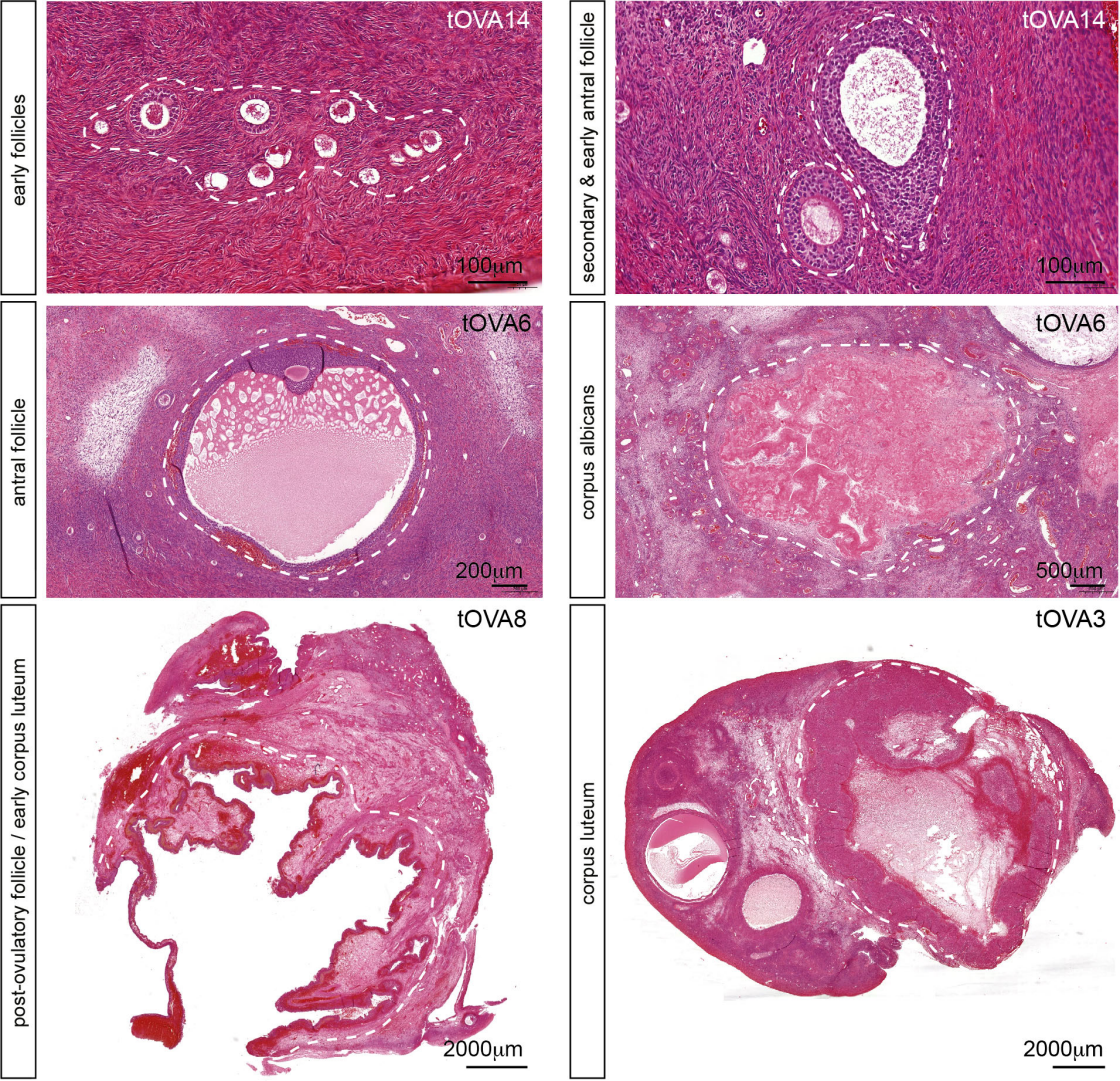
Folliculogenesis in transmasculine ovaries. Hematoxilin-eosin staining on ovarian histological sections from several transmasculine donors showing different follicular stages, such as early follicles (primordial, transitional, primary and early secondary), secondary and early antral follicles, antral follicles, post-ovulatory follicles (or early corpus lutea), corpus lutea and corpus albicans. Scale bars are indicated.

In this study, the population of transmasculine donors (**Table S1**) showed ovaries in the follicular phase, but surprisingly also in the luteal phase after histological analysis (**Figure 1**), suggesting that active folliculogenesis is taking place at least in some donors. Although this may be a sporadic observation, it contrasts with the suggestion that testosterone treatment may suppress ovulation in transmasculine donors (35). More importantly, this suggests that ovaries from transmasculine donors may be adequate to investigate folliculogenesis *in vitro*.

### Distribution and characterization of follicular stages in cortical ovarian fragments

**Figure 2:**
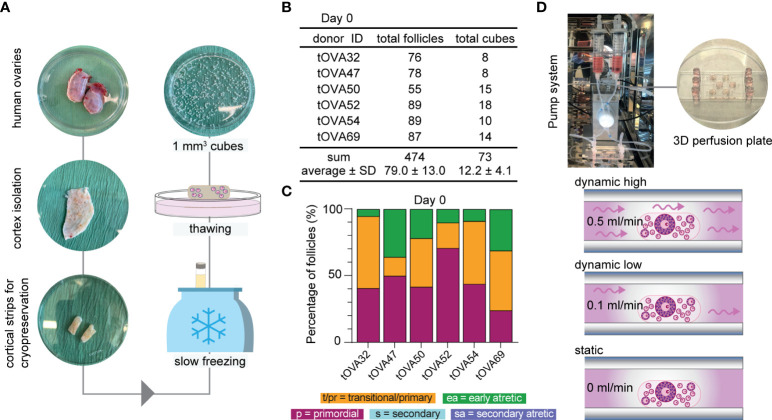
Study design and follicular distribution at D0. **(A)** Schematic workflow from collection until culture. Upon arrival, the ovaries were bisected and the outer cortex was isolated by scraping the medulla and inner cortex. Cortical strips were prepared for cryopreservation using slow freezing. Thawed-cortical tissue was cut into approximately 1mm3 cubes to fix or culture. **(B)** Total number of follicles and cubes analyzed per donor at D0, sum and average ± standard deviation (SD). **(C)** Follicular distribution per donor at D0. **(D)** Ovarian cortical cubes were placed in microwells (1-2/well) from μ-slide III 3D perfusion plates. Microchannels were filled with culture medium and plates were either connected to the perfusion pump for dynamic culture applying high flow rate (0.5ml/min), low flow rate (0.1ml/min) or no flow (0ml/min).

Cryopreserved-thawed cortical ovarian fragments from 6 different donors undergoing gender-affirming surgery (**Table S1**) were cut into cubes of approximately 1mm^3^ size (**Figure 2A**) and fixed at day 0 (D0) for baseline follicular population analysis. A total of 73 cubes containing 474 follicles were analyzed at D0 and showed an average of 79.0 ± 13.0 follicles per donor (**Figure 2B**). The distribution of the different follicular stages was 45.2 ± 6.2% primordial follicles, 35.9 ± 6.6% primary and transitional follicles, 18.9 ± 5.2% early atretic follicles, and no secondary follicles (healthy or atretic) (**Figure 2C** and **Figures S1A–C**). Cryopreserved-thawed cortical cubes were placed into the Ibidi III 3D perfusion system and cultured for 4 days (D4) or 8 days (D8) under different flow rates (0.5 ml/min; 0.1 ml/min; 0 ml/ml) (**Figure 2D**).

**Figure 3:**
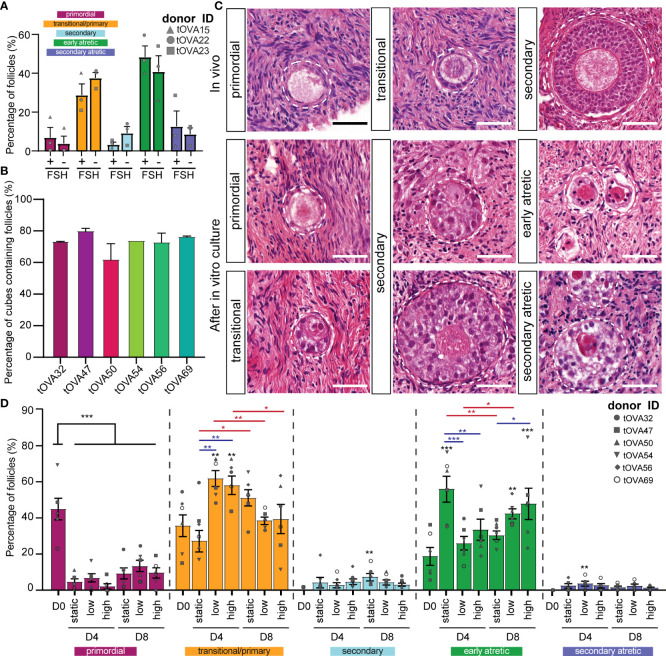
Distribution of follicles in ovarian cortical cubes after *in vitro* culture. **(A)** Distribution of follicular stages after eight days in culture with or without 1ng/ml of FSH. Results depict the percentage of each follicular stage per condition (mean ± SEM) compared per follicular stage. A total of N=120 cortical tissue cubes containing in total 321 follicles were classified. Statistical analysis was performed using Shapiro-Wilk test for normal distribution followed by multiple t-test. *=p_value < 0.05; ** = p_value < 0.01; ***=p_value <0.001. **(B)** Percentage of cortical tissue cubes containing follicles per donor (mean ± SEM). **(C)** Follicular classification used in this study. Top row shows *in vivo* control ovarian follicles in histological sections from freshly-isolated transmasculine donors. Bottom rows show *in vitro* cultured ovarian follicles. Scale bars = 40 μm. **(D)** Distribution of follicular stages after D4 and D8 in static, dynamic low and dynamic high culture. Results depict the percentage of each follicular stage per condition (mean ± SEM) compared per follicular stage. A total of N=497 cortical tissue cubes containing in total 3676 follicles were classified. Statistical analysis was performed using one-way ANOVA followed by Fisher test comparing each condition to D0 and statistical significance visualized on top of each respective bar (*=p_value < 0.05; ** = p_value < 0.01; ***=p_value <0.001). Statistical analysis was also performed among the three culture conditions within the same time point (blue lines) and between D4 and D8 within the same condition (red lines).

The culture medium used was essentially as previously published (33, McLaughlin et al., 2018) with the main differences that FSH was not added. The reason being that 1) the activation of primordial follicles is independent of FSH (17) and 2) we observed no statistical differences in the distribution of follicular types after culturing fresh cortical ovarian cubes in static condition for eight days in the presence or absence of FSH (**Figure 3A**).

Histological analysis of the cryopreserved-thawed cortical cubes showed remarkable variation in follicular distribution after culture. Moreover, it is noteworthy that many fragments from all 6 donors were empty of any type of follicles (**Figure 3B** and **Figure S1A**), in agreement with reports showing that the human ovarian cortex contains an uneven follicular distribution (36–38), hence it is important to analyze a large number ovarian cortical cubes.

As in freshly isolated ovaries (*in vivo*), we observed all stages of preantral follicles after culture (**Figure 3C**). Regarding the morphology of the follicles after culture, primordial follicles containing a single layer of flat granulosa cells could be distinguished from follicles that showed the presence of cuboidal granulosa cells, often surrounding the oocyte only partially (**Figure 3C**). As from one single section it was not possible to distinguish between primary follicles (completely surrounded by cuboidal granulosa cells) and transitional follicles (partially surrounded by cuboidal granulosa cells), we quantified those into a single group (primary and transitional follicles). Furthermore, two types of secondary follicles could be distinguished after culture: one type with a centrally located oocyte (**Figure 3C** middle- bottom) and another with a peripherally located oocyte (**Figure 3C** middle-top). Both types were quantified together as secondary follicles. The atretic follicles were distinguished by an oocyte with a red cytoplasm and characteristic pyknotic nucleus also observed in the surrounding granulosa cells (**Figure 3C**).

### Influence of the flow rate in the culture of human cortical tissue

Compared to the follicular distribution at D0, all conditions showed a significant decreased in the percentage of primordial follicles (p_value < 0.001) displaying 4.6 ± 1.7% at D4 static; 9.1 ± 3.2% at D8 static; 6.7 ± 2.4% at D4 dynamic low; 13.2 ± 3.4% at D8 dynamic low; 2.1 ± 1.5% at D4 dynamic high; and 9.6 ± 2.8% D8 dynamic high (**Figure 3D** and **Figures S1B–D**).

After four days of culture, the percentage of primary/transitional follicles obtained in the absence of flow 33.3 ± 6.1% was comparable to that observed at D0, but the number of early atretic follicles (55.9 ± 7.1%) was significantly higher than that observed at D0 (p_value=0.0001). This suggested that this culture condition was not beneficial for folliculogenesis. By contrast, the ovarian cubes exposed to flow (low and high) exhibited a significant increase in the percentage of primary/transitional follicles (61.6 ± 4.4% and 57.9 ± 5.1%, respectively), compared to D0 (p_value=0.003 and p_value=0.009, respectively) (**Figure 3D** and **Figures S1B–D**). Moreover, several secondary follicles were observed after four days of culture, but the number was relatively low in all culture conditions (**Figure 3D** and **Figures S1B–D**). Together, this suggested that exposure to flow during four days stimulated follicular activation.

Interestingly, the ovarian cubes cultured for eight days without flow showed the highest percentages of both primary and transitional (51.0 ± 4.6%) and secondary follicles (7.0 ± 1.9%) from all culture conditions (**Figure 3D** and **Figures S1B–D**). Moreover, the increment in secondary follicles after eight days of culture was only statistically significant in the static condition when compared to D0 (p_value=0.006). It is important to note that the total number of secondary follicles obtained after culture was low in all conditions (**Figure S1B**).

Together our results suggest that the use of flow may accelerate follicular activation until four days of culture, but may not be beneficial for further follicular growth at least in combination with the used culture media.

### Characterization of the granulosa cells in the secondary follicles after culture

**Figure 4:**
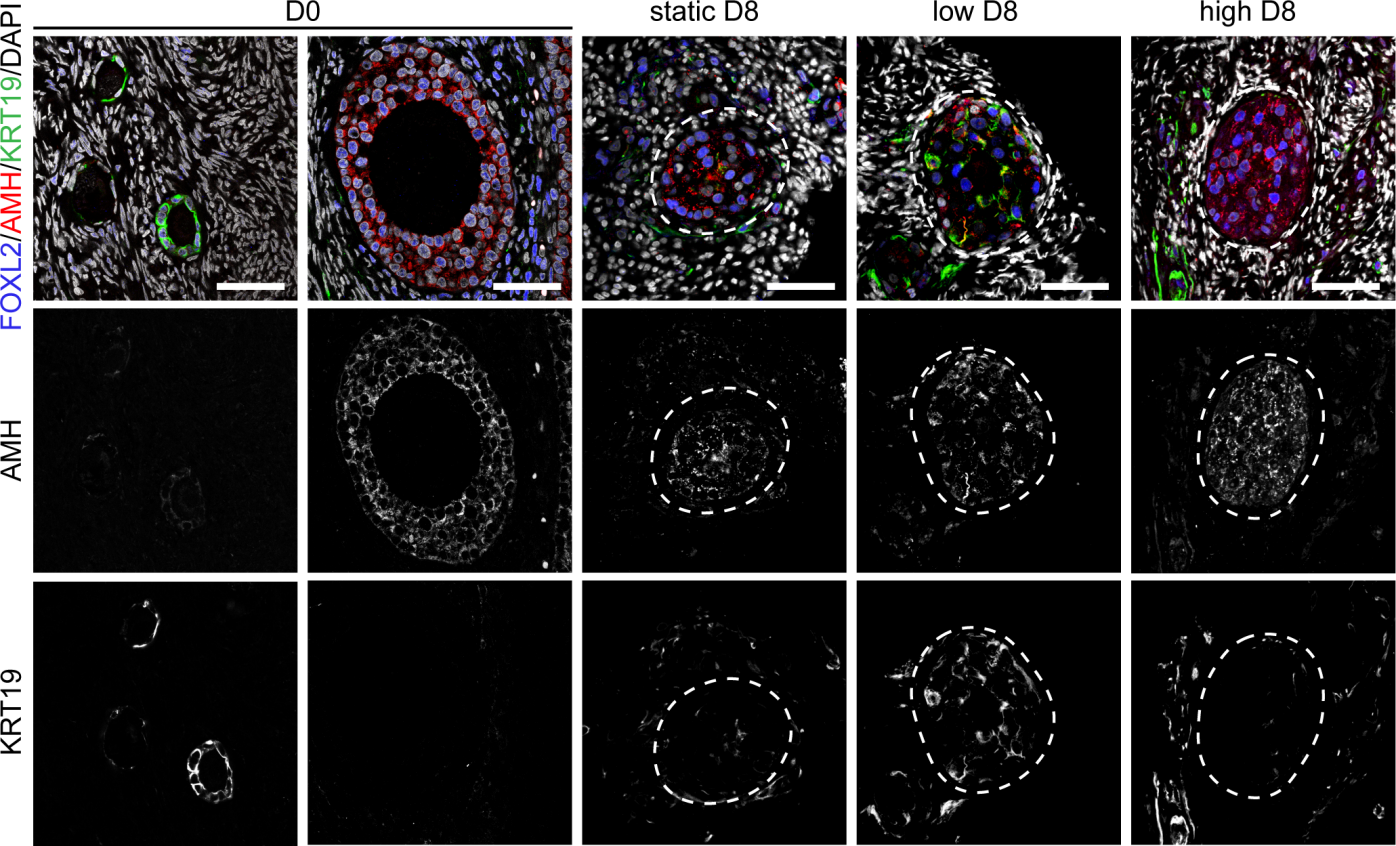
Expression of FOXL2, AMH and KRT19 in secondary follicles after culture. Immunofluorescence for FOXL2, AMH and KRT19 in primordial, primary and secondary follicles at D0 and after eight days (D8) culture in static, low and high flow rate. Scale bars = 50 μm.

To further investigate the quality of the secondary follicles obtained after culture under different flow rates, we performed immunofluorescence for anti-Müllerian hormone (AMH) and keratin 19 (KRT19) (**Figure 4** and **Figure S2**). AMH is a member of the TGFβ-family involved in the regulation of folliculogenesis and expressed in human ovarian follicles from the secondary stage onwards (39) (**Figure 4**). KRT19 belongs to the keratin family of intermediate filaments, with a main role in the structural integrity of epithelial cells. The expression of KRT19 in preantral follicles in humans has not been previously reported, however we observed specific expression of KRT19 in granulosa cells of unilaminar follicles, but not in granulosa cells of secondary follicles at D0 (**Figure 4**). Hence, we considered the dynamic expression pattern of AMH and KRT19 suitable to assess the transition from unilaminar to secondary follicles after culture.

After four and eight days of culture, we observed an increased disorganization in the granulosa cells of secondary follicles, marked by the transcription factor forkhead box L2 (FOXL2), when compared to the granulosa cells in non-cultured secondary follicles (**Figure 4** and **Figure S2**). After four days in culture, granulosa cells in secondary follicles showed both expression of AMH and KRT19. By contrast, after eight days in culture the granulosa cells of the secondary follicles cultured under low flow rate showed both expression of AMH and KRT19, whereas the granulosa cells of the secondary follicles cultured under high flow rate or static showed expression of AMH and absence of KRT19 (**Figure 4**). This suggested that the granulosa cells present in the secondary follicles after eight days in culture under low flow rate still retained characteristics of those in unilaminar follicles.

### Cell viability and proliferation of the granulosa cells in the secondary follicles after culture

To study the viability of the cultured follicles and surrounding stromal tissue, the TUNEL assay was used to detect apoptosis through DNA fragmentation. Moreover, we evaluated the presence of a continuous uninterrupted follicular basement membrane using immunofluorescence for collagen type IV (COLIV). The basement membrane surrounding the ovarian follicles is rich in extracellular matrix proteins, such as COLIV (40) and plays a fundamental role on follicular survival, since its disruption compromises follicular viability (41, 42).

**Figure 5:**
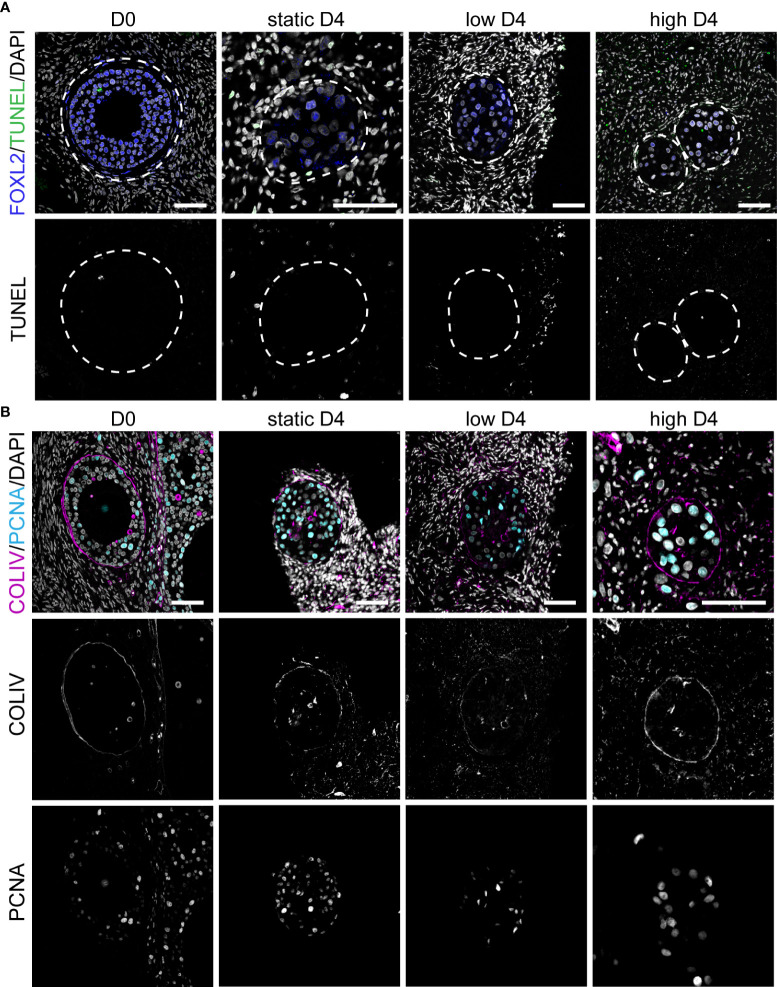
Apoptosis, proliferation and COLIV deposition in ovarian cortical cubes after four days of culture. **(A)** Immunofluorescence for FOXL2 together with TUNEL assay in secondary follicles at D0 and after four days (D4) culture in static, low and high flow rate. Scale bars= 50 μm. **(B)** Immunofluorescence for COLIV and PCNA in secondary follicles at D0 and after D4 culture in static, low and high flow rate. Scale bars= 50 μm.

**Figure 6:**
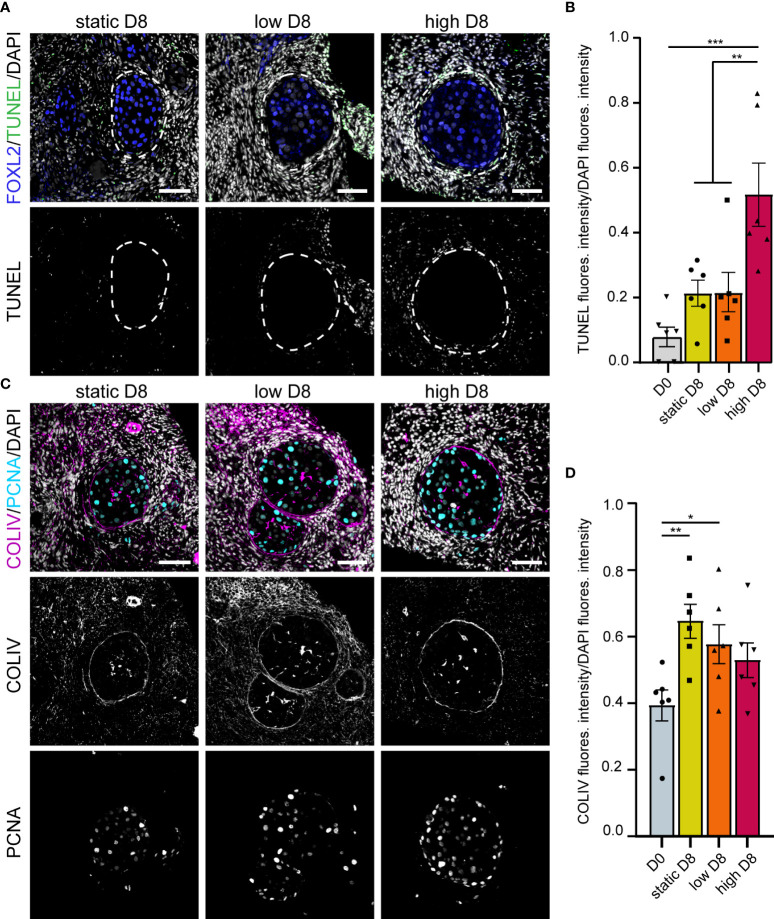
Apoptosis, proliferation and COLIV deposition in ovarian cortical cubes after eight days of culture. **(A)** Immunofluorescence for FOXL2 together with TUNEL assay in secondary follicles after eight days (D8) culture in static, low and high flow rate. Scale bars= 50 μm. **(B)** Relative TUNEL fluorescence intensity in ovarian cortical cubes at D0 and after D8 culture in static, low and high flow rate. Statistical analysis was performed using one-way ANOVA followed by Fisher test (** = p_value < 0.01; (*** = p_value < 0.001). **(C)** Immunofluorescence for COLIV and PCNA in secondary follicles after D8 culture in static, low and high flow rate. Scale bars= 50 μm. **(D)** Relative COLIV fluorescence intensity in ovarian cortical cubes at D0 and after D8 culture in static, low and high flow rate. Statistical analysis was performed using one-way ANOVA followed by Fisher test (* = p_value < 0.05; ** = p_value < 0.01).

After four days in culture, secondary follicles showed no TUNEL+ follicular cells. Only the stromal cells at the edge of the cubes showed signs of apoptosis (**Figure 5A**), perhaps due to tissue damage during sample preparation. Furthermore, all secondary follicles exhibited an intact COLIV+ basement membrane, as well as proliferative PCNA+ granulosa cells, comparable to that observed in D0 (**Figure 5B**). After eight days in culture, there were still no double positive FOXL2+TUNEL+ granulosa cells in all culture conditions (**Figure 6A**). However, there was a statistically significant increase in TUNEL+ cells in the stromal compartment of the ovarian cubes under high flow compared to both DO (p<0.0001) and to the other D8 conditions (p=0.001) (**Figure 6B**). Interestingly, the levels of proliferation (PCNA) observed after eight days in culture were opposite to those of TUNEL, with high levels of PCNA in the granulosa cells of secondary follicles whereas the stroma was mostly PCNA-negative (**Figure 6C**).

Secondary follicles obtained after eight days of culture showed a continuous COLIV+ basement membrane, comparable with that of secondary follicles at D0 (**Figure 6C**). In addition, the deposition of COLIV between the granulosa cells (Call-Exner bodies) (43) was also present in the secondary follicles in all culture conditions. Interestingly, after eight days in culture, we observed a statistically significant increase in COLIV deposition in the stromal compartment of the ovarian cubes cultured with low or no flow (p_value=0.004 and p_value=0.03, respectively), when compared to ovarian cubes at D0 (**Figure 6D**). It remains to be investigated whether the higher levels of COLIV deposition on the stromal compartment have a detrimental effect on follicular growth in long-term culture.

## Discussion

Microfluidic devices including dynamic culture conditions have proved to be an innovative system for creating a more physiological microenvironment during *in vitro* culture of embryos (44), ovarian cells (30), ovarian cortical tissue and isolated follicles from animal models (45). Here, we aimed to test whether the culture of human ovarian cortical tissue under dynamic conditions for eight days improved activation, growth and viability of early follicles. We used human ovarian cortical samples that have been cryopreserved-thawed following clinical-grade protocols to increase applicability.

*In vivo* folliculogenesis from primordial to pre-ovulatory follicle takes several months in humans (16, 17), but it has been shown that *in vitro* this process is strongly accelerated (26, 27). De Roo and colleagues have proposed that mechanical fragmentation of the cortical tissue during sample preparation for *in vitro* culture disrupts the Hippo pathway, promoting the activation of dormant follicles as well as the growth of other follicular stages (46). This could explain the formation of preantral follicles in such a short period of culture. Moreover, we have included a short-term treatment with S1P, a follicular growth-promoting lipid that is involved in both the Hippo- and PI3K- pathways (33) and could therefore promote and accelerate *in vitro* folliculogenesis.

The quantification of the follicles in cryopreserved-thawed cortex samples from gender-affirming donors at D0 already revealed that an average of 19% of the follicles were atretic, a much higher percentage than that reported in cisgender young donors (47). As reported in non-human primates (48), a detrimental effect from the androgen treatment on follicular quality in the transmasculine ovarian samples at D0 cannot be excluded, although De Roo and colleagues reported both a comparable follicular distribution and *in vitro* maturation potential of oocytes isolated from cumulus-oocyte-complex between androgen-treated and untreated ovaries (49). Alternatively, the cryopreserved-thawing procedure could directly affect the percentage of atretic follicles at D0.

The number of secondary follicles that was formed in cryopreserved-thawed ovarian cortical cubes after eight days of culture in both static and dynamic conditions was low. Moreover, although the secondary follicles obtained showed an intact basement membrane and their granulosa cells showed proliferative capacity and production of AMH, suggesting both viability and functionality, the granulosa cells showed in general a high level of cellular disorganization in the follicle that resulted in the peripheral location of the oocyte. In agreement, Wang and colleagues showed that a larger number of abnormal follicles was observed as well as significantly lower expression of follicular markers, as ZP3, CYP11A and AMH in ovarian tissue after cryopreservation, slow freezing and vitrification, compared to fresh counterparts after culture (50). By contrast, work by Sanfilippo and colleagues did not find significant differences regarding the percentage of viable follicles from frozen-thawed versus fresh ovarian cortical tissue, and they reported 20% of secondary follicles in cryopreserved fragments after culture (51). In order to validate the *in vitro* growth capacity of the cryopreserved-thawed cortical tissue obtained from transmasculine donors, a fresh age-matched control group should be considered in future experiments.

The presence of apoptotic cells in the stroma deserves special attention. Sanfilippo and colleagues reported a higher amount of TUNEL+ stromal cells on ovarian cortical tissue induced by cryopreservation-thawing (51). The correlation between stromal cell function and follicular growth and survival have been previously investigated (52). Qui and colleagues carried out an *in vitro* assay involving the co-culture of granulosa cells with stromal/theca cells from goat ovaries to evaluate the effect of factors secreted by the stromal cells in the survival and functionality of granulosa cells. It was found that stromal/theca cells promoted granulosa cells proliferation and improved their viability by triggering pro-survival BCL2 gene expression and inhibiting the production of pro-apoptotic BAX gene and CASP3 activation (52). Therefore, increased levels of apoptosis in the stroma may be highly detrimental to the follicular growth and follicular quality. The fact that many stroma cells were TUNEL+ indicated that this cellular compartment showed high sensibility to the *in vitro* culture conditions tested. By contrast, the granulosa cells seemed to be proliferative and produced AMH, reflecting less sensitivity, but because they showed disorganization and retained expression of KRT19, this may reflect a defective development.

After eight days in culture, in all the conditions investigated (static, dynamic low, dynamic high) the ovarian cubes showed a strongly collagenized stroma, with high deposition of COLIV, when compared to D0. High collagenization has been reported in aged ovaries of non-human primates, containing both lower follicular density and a higher number of atretic follicles (53). It seems unquestionable that the stroma plays a major role in the regulation of folliculogenesis, hence it is likely that both higher stromal apoptosis and collagenization may negatively affect follicular growth *in vitro* and consequently result in low number of secondary follicles. A possible reason for this is the composition of the culture medium, that may be suitable to culture fresh ovarian cortex, but not adequate to culture cryopreserved-thawed ovarian cortex, that may have an altered metabolic activity or decreased mitochondrial activity (54). Moreover, as the ovaries of transmasculine donors are exposed to an abrupt decrease in the levels of testosterone, they may not respond adequately to the culture medium used.

Although cryopreserved-thawed ovarian cortical tissue has proved viable and functional when retransplanted into the patient’s body by restoring both endocrine ovarian function and fertility (3, 4), their use for *in vitro* folliculogenesis remains challenging. The culture of ovarian cortical tissue within microwells in a dynamic culture system may offer advantages compared to the traditional static culture, such as a more physiologically relevant supply of culture media and a reduction in manipulation. However, the application of a dynamic culture system to support *in vitro* folliculogenesis successfully will require several major improvements to accelerate optimization. First, the use of a higher number of ovarian cubes is desirable to obtain enough secondary follicles to allow a deeper characterization of the *in vitro* culture system. In order to do it, multiple μ-slide III 3D perfusion plates should be interconnected in series and perfused by a single pump. Secondly, thinner cortical fragments or the use of a follicular vital dye, such as Neutral Red (55) should be considered to exclude cortical fragments empty of follicles from culture.

To focus on follicular distribution rather than on the total number of follicles, we performed analysis on the percentage of follicle types per donor per condition and used the sum of follicles in all cubes per donor to eliminate the variation between cubes per condition. Nevertheless, we cannot exclude that some of the variation detected is caused by the inner variability among donors or between cubes. To overcome this aspect, it is important to use both a high number of cubes and a high number of donors.

In conclusion, we report that the culture of cryopreserved-thawed ovarian cortical tissue fragments under static condition proved to be a more efficient method to obtain secondary follicles after eight days in culture when compared to both the low and high dynamic conditions tested. Moreover, high flow rates seemed detrimental for the tissue quality. Ovarian cortical tissue cubes cultured under static condition showed low TUNEL levels in both follicular and stromal cells, displaying a higher potential for being cultured for a longer period of time. Nevertheless, the total number of secondary follicles obtained under static culture remained very low, indicating that additional studies are required to elucidate the culture conditions that can lead to efficient follicular growth *in vitro*.

## Data availability statement

The raw data supporting the conclusions of this article will be made available by the authors, without undue reservation.

## Author contributions

JD, VM, NM, and SC conceived the study. JD, VM, JA, XF, NM, LL, GP, and LW contributed to material collection. JM, JA, NM, and SC arranged the ethical permit. JD, VM, and ML generated data. All authors contributed to data analysis. All authors contributed to manuscript writing. All authors approved the submitted version.

## Funding

This research was funded by the European Research Council (OVOGROWTH ERC-CoG-2016-725722 to JD, VM, XF and SC.

## Acknowledgments

We would like to thank all patients that donated tissue for this study and the members of the Chuva de Sousa Lopes group for useful discussions.

## Conflict of interest

The authors declare that the research was conducted in the absence of any commercial or financial relationships that could be construed as a potential conflict of interest.

## Publisher’s note

All claims expressed in this article are solely those of the authors and do not necessarily represent those of their affiliated organizations, or those of the publisher, the editors and the reviewers. Any product that may be evaluated in this article, or claim that may be made by its manufacturer, is not guaranteed or endorsed by the publisher.
