# Supplementary material for: Dynamic in vitro culture of cryopreserved-thawed human ovarian cortical tissue using a microfluidics platform does not improve early folliculogenesis
Source: Front Endocrinol (Lausanne). 2022 Jul 29;13:936765. doi: 10.3389/fendo.2022.936765 (PMC9372461; doi:10.3389/fendo.2022.936765)
Supplement: Supplementary file 1 [file DataSheet_1.docx]

**Supplementary Material**

**Dynamic in vitro culture of cryopreserved-thawed human ovarian cortical tissue using a microfluidics platform does not improve early folliculogenesis**

Julieta S. del VALLE^1^, Vanessa MANCINI^1^, Maitane LAVERDE GARAY^1^, Joyce D. ASSELER^2^, Xueying FAN^1^, Jeroen Metzemaekers^3^, Leoni A. LOUWE^3^, Gonneke S. K. PILGRAM^3^, Lucette A. J. van der WESTERLAKEN^3^, Norah M. van MELLO^2^, Susana M. CHUVA DE SOUSA LOPES^1,4,*^

^1^ Department of Anatomy and Embryology, Leiden University Medical Center, Leiden, Netherlands

^2^ Amsterdam UMC, Department of Obstetrics and Gynaecology, Amsterdam, The Netherlands

^3^ Department of Gynaecology, Leiden University Medical Center, Leiden, The Netherlands

^4^ Ghent-Fertility and Stem Cell Team(G-FAST), Department of Reproductive Medicine, Ghent University Hospital, Ghent, Belgium

* Corresponding Author: Susana M. CHUVA DE SOUSA LOPES, email: lopes@lumc.nl

**Contents:**

- **Table S1**
- **Figure S1**
- **Figure S2**

**Table S1. Characteristics of the ovarian cortical tissue donors**

| **Donor ID** | **Age**  **(years)** | **Type of gender affirming hormone therapy** | **Duration of gender affirming hormone therapy (months)** |
| --- | --- | --- | --- |
| tOVA3 | 19 | Sustanon | 22 |
| tOVA6 | 20 | Sustanon | 30 |
| tOVA8 | 24 | Androgel | 35 |
| tOVA14 | 18 | Sustanon | 22 |
| tOVA15 | 26 | Sustanon | 41 |
| tOVA22 | 24 | Nebido | 60 |
| tOVA23 | 24 | Androgel | 56 |
| tOVA32 | 21 | Nebido | 23 |
| tOVA47 | 41 | Androgel | 32 |
| tOVA50 | 26 | Sustanon | 47 |
| tOVA52 | 24 | Nebido | 60 |
| tOVA54 | 25 | Androgel | 52 |
| tOVA69 | 30 | Sustanon | 66 |

**
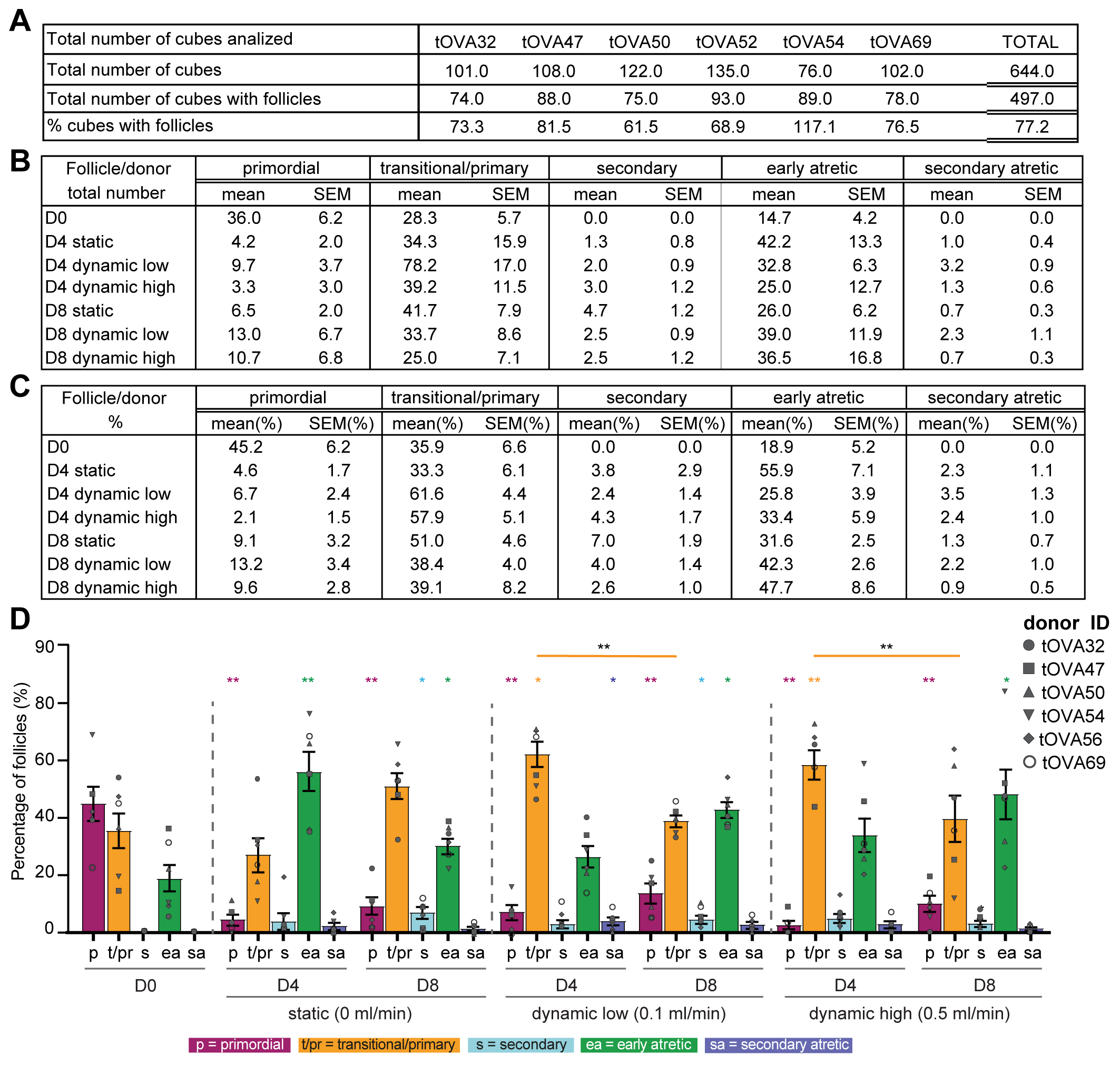
**

**Figure S1. Quantification of follicular distribution in ovarian cortical cubes.**

**(A)** Total number of cubes analysed and percentage of cubes with follicles per donor.

**(B)** Mean total follicle number and SEM of the different follicles stages per condition.

**(C)** Mean average and SEM of the different follicles stages per condition.

**(D)** Distribution of follicular stages after D4 and D8 in culture from static, dynamic low and dynamic high culture. Results depict the percentage of each follicular stage per condition (mean ± SEM) compared per follicular stage. A total of N=497 cortical tissue cubes containing in total 3676 follicles were classified. Statistical analysis was performed using two-way ANOVA followed by Fisher test comparing each follicular stage after culture to D0 with the statistical significance visualized on top of each respective bar. Statistical analysis was also performed between D4 and D8 within each culture condition only from the same follicular stage (black lines). (*=p_value < 0.05; ** = p_value < 0.01; ***=p_value <0.001).


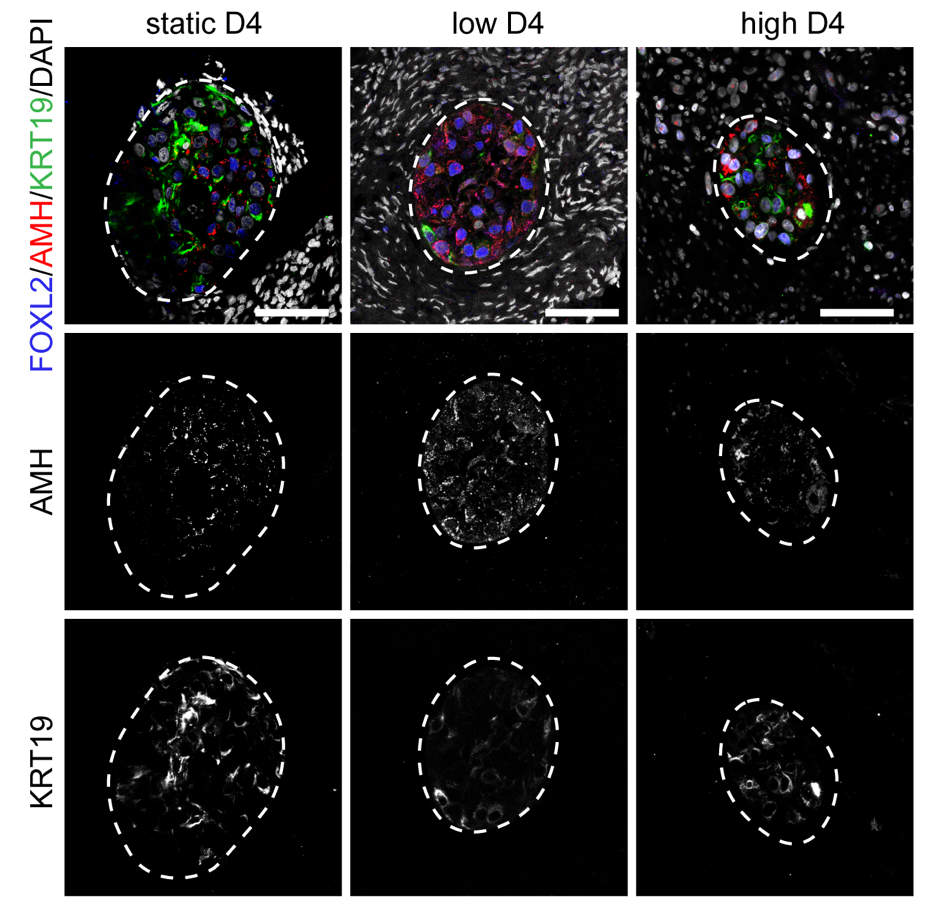


**Figure S2. Expression of FOXL2, AMH and KRT19 in secondary follicles after four days in culture.** Immunofluorescence for FOXL2, AMH and KRT19 in secondary follicles after four days of culture in static, low and high flow rate. Scale bars= 50 μm.
